# Examining health equity in Nepal’s climate change and health policies through the lens of environmental justice: insights from a content analysis

**DOI:** 10.1080/16549716.2024.2432069

**Published:** 2024-11-29

**Authors:** Sudeepa Khanal, Rehana Shrestha, Melanie Boeckmann

**Affiliations:** a Faculty of Health Sciences, University of Bielefeld, Bielefeld, Germany; b Department of Social Epidemiology, Institute of Public Health and Nursing Research, University of Bremen, Bremen, Germany; c Department of Global Health, Institute of Public Health and Nursing Research, University of Bremen, Bremen, Germany

**Keywords:** Environmental health equity, climate change policy, public health policy, policy research, environment justice, content analysis, Nepal

## Abstract

**Background:**

Climate change presents a multifaceted challenge with unequal health implications, particularly for vulnerable populations with limited adaptive capacity. Socioeconomic factors are intricately linked with environmental health outcomes and environmental factors significantly exacerbate existing health inequities. Health equity as a goal of environmental justice can address these issues.

**Objective:**

To examine the integration of health equity within climate change and health policy documents in Nepal.

**Methods:**

Using a qualitative content analysis approach based on Schlosberg’s framework of environmental justice, we analyzed the coverage of health equity considerations in climate and health policies, assessing aspects of distribution, recognition, and participation.

**Results:**

Twenty-one national-level policies, strategies, and plans/guidelines on climate change and health were analyzed. Nepal’s policy documents lack clear definitions of health equity in relation to climate change, and related terms are used inconsistently. Health vulnerability is often addressed broadly rather than specifically. Health equity-related statements from environmental justice viewpoint vary across sectors. Many documents emphasize equitable distribution of resources and benefits, with participation in decision-making processes being the least discussed.

**Conclusions:**

In Nepal, lack of shared understanding of health equity across sectors hinders coordinated policy efforts. There is an urgent need to expand climate change responses to consider specific health vulnerabilities. By positioning health equity as a key element of environmental justice, this study provides a broader perspective on climate change-related health equity that could encourage collaborative action between the environment and health sectors.

## Paper Context

- **Main Findings**: Analysis of the policy documents in Nepal reflects the recognition of the need to integrate health equity into climate change policies comprehensively by various ministries due to their interconnected nature. However, when assessed through the three principles of environmental justice, health equity considerations vary significantly across sectors, each emphasizing different principles and to varying degrees.
- **Added Knowledge**: To our knowledge, this study is the first to explore health equity considerations in climate change policies in the context of Nepal. This is also the first study to use an environment justice lens to understand health equity in the realm of climate change in a low- and middle-income country.
- **Global health impact for policy and action**: This study attempts to bridge the gap between the health and the environmental sectors by proposing climate change-related health equity as an environmental justice issue and drawing on their linkages. It also provides a theoretical-based policy analysis framework for various public health and climate change researchers seeking to understand this complex landscape. In Nepal’s evolving federalized context, understanding health equity considerations in existing national policies can facilitate advocating for vulnerable groups and mainstreaming health equity into climate policies across all government levels by providing transferable insights.

## Background

Climate change significantly contributes to health inequity due to the close connection between health and the environment [1]. Addressing both environmental hazards and social conditions is crucial for reducing climate change-related health disparities, especially in vulnerable communities [2,3]. Health equity refers to the condition where everyone has a fair chance to achieve their full health potential without being disadvantaged by their social, economic, or environmental circumstances [4]. The role of various environmental factors in exacerbating existing health inequalities has been well recognized. Environmental conditions are major determinants of health and well-being, but they vary for individuals and communities. Inequities in environment determinants impact health equity in four distinct ways: 1. by leading to uneven exposure to unhealthy environments, 2. shaping individual behaviors that affect exposure levels and health outcomes, 3. increasing vulnerability to environmental factors negatively impacting health and well-being, and 4. potentially resulting in limited access to services that could alleviate the effects of unhealthy environmental exposures [5]. Given the links between socio-economic factors and environmental health disparities, the concepts of environmental equity, environmental justice and climate justice are becoming increasingly prominent in discussions regarding health equity and climate change [6].

Climate change is an environmental justice concern, as it obstructs the right to optimal health [7]. Health impacts of climate change can be positioned within the larger contextual socioeconomic and environmental frameworks, and unequal distribution of health risks can be analyzed using concepts of equity and environmental justice. While environmental justice and health equity are interrelated concepts and achieving one requires consideration of the other, how they fit in the overall climate change-related policies needs further exploration.

‘Environmental equity’ emphasizes fair distribution of environmental resources and burdens, distinguishing between outcome equity (distribution of environmental advantages and disadvantages among affected populations), and process equity (fairness of decision-making processes) [8]. ‘Environmental justice’ however broadens this concept by advocating for the right of all individuals and communities to a healthy environment. It seeks to address environmental inequalities and injustices through policy advocacy, community organizing, and legal action, particularly focusing on the disproportionate impacts of environmental hazards and pollution on marginalized communities [9,10]. Bolte et al. define environmental justice as more than just social inequalities linked to health and the environment, proposing a focus on two key components distributional justice and procedural justice [11]. Whereas, Schlosberg argues that environmental justice entails three fundamental principles: equitable distribution of environmental risks, recognition of the diverse backgrounds and viewpoints within affected communities, and participation in the political processes governing environmental policies [12].

Given that most existing health equity policy tools and frameworks primarily focus on evaluating the effectiveness of climate change-related policies based on health equity outcomes, rather than assessing how well these policies incorporate health equity considerations, using Schlosberg’s three principles of environmental justice to understand health equity considerations can underscore the intersectionality of climate change-related health equity issues within the realms of environmental justice. This approach helps conceptualize health equity holistically, beyond the disproportionate health impacts of climate change.

## Climate change in Nepal

Nepal, a federal democratic republic in South Asia, faces significant challenges due to its vulnerability to climate change driven by its challenging geography and poor socio-economic conditions [13]. With a population of approximately 29 million and a ranking of 145th on the Human Development Index, Nepal also ranked tenth on the global climate risk index 2021 [14]. The country has experienced consistent warming and increased maximum temperatures leading to numerous climate change impacts and health consequences, particularly in high-altitude regions. Waterborne, vector-borne, air-borne, and food-borne illnesses, along with malnutrition and injuries, are prominent health consequences observed in the country. Nepal struggles with health system inequities exacerbated by climate change [15]. The Constitution of Nepal guarantees the right to a clean environment as well as equal access to healthcare, reflecting the concepts of both environmental justice and health equity [16]. The GoN has developed a number of significant climate change specific policies, both mitigation and adaptation, aimed at guiding various levels in building a resilient society by reducing climate change impacts [17]. These include the Initial National Report to the UNFCCC (2004), the National Adaptation Plan of Action (NAPA − 2007), the Local Adaptation Plan of Action (LAPA − 2010), the REDD Readiness Preparedness Proposal (2010), and the Nepal Climate Change Policy (2011 and 2019), National Adaptation Plan (NAP) 2021–2050, among others [18]. Under the new federal structure, the Ministry of Forest and Environment [15] is the focal point for climate change-related issues, coordinating with several ministries and international protocols [17].

The Government of Nepal (GoN) has identified public health as one of the sectors most vulnerable to the negative effects of climate change. Similarly, the Ministry of Health and Population (MoHP) also recognizes climate change as a major threat to the health system and stresses the importance of addressing climate change-related health inequities, as reflected in National Health Policy 2014 and 2019, Nepal Health Sector Strategy (2015–2020), and Nepal Health Sector Strategic Plan (2022–2030) [18,19]. As part of its efforts, the MoHP has carried out a comprehensive climate change vulnerability and risk assessment and developed the Health National Adaptation Plan (H-NAP 2016–2020) in accordance with international guidelines and standards. However, the efforts have largely focused on the development of the H-NAP, and several challenges persist for its implementation. There are also limited information available regarding integration of health equity and climate change considerations beyond this document [18].

Nepal, like many other low and middle-income countries, struggles to address climate change-related health disparities due to variety of factors, including financial constraints [20]. While Nepal acknowledges the unequal impact of climate change on its population, there is limited exploration of policy priorities to address health disparities. As is the case elsewhere, most of the available studies largely focus on understanding the impact of climate hazards on health. A systematic review of literature on climate and health vulnerability in Nepal since 2010 identified 37 studies on climate change vulnerabilities [21], however, none of the evidence was on the current state and progress on integrating climate change and health policies. Assessing the integration of health equity in national health and climate policies is crucial for effectively addressing climate-related health inequities. To comprehensively assess health equity in climate change-related policies from an environmental justice standpoint, it’s vital to examine how health equity considerations within broader climate and health policies align with the principles of environmental justice.

## Aim

This study aimed to examine the coverage of health equity within climate change and health policies and strategies in Nepal through an environmental justice lens. We assessed the content of existing climate change-related policies, strategies, and guidelines documents at the federal level in Nepal.

## Objectives

The study was guided by two primary questions:

In Nepal,

1. how is health equity in relation to climate change defined in the (policy/strategy) documents?
2. how is health equity described in reference to the concepts of environmental justice?

## Methods

### Analysis framework

To systematically analyze health and climate change policy documents for their prioritization, operationalization, and integration of health equity, Schlossberg’s framework of environmental justice was used. This framework encompasses three important principles: distribution, recognition, and participation, which are crucial for assessing health equity both generally and in the context of climate change. A comprehensive coding frame was developed, categorizing health equity considerations in the policy text into the three environmental justice principles: distribution, recognition, and participation.

#### Documents identification

We systematically reviewed two types of documents to gain insights into health equity from the perspective of environmental justice and climate change. A) The constitution, acts/regulations, and overarching national documents that frame the social and political context for climate change-related policies. B) Current federal-level health and climate change policies, acts, key strategies, and action plans of MoHP and MoFE that have been officially endorsed. The H-NAP document from the MoHP was excluded because our focus was to explore integration beyond this specific document. National reports on climate change were excluded from the study as our aim was to understand policy integration rather than data utilization in policymaking. Similarly, policies from ministries other than MoHP and MoFE were excluded to maintain a focus on health equity.

Where available, we accessed the official translated English versions of the documents. These were retrieved from the websites of GoN, MoHP, and MoFE. If not available online, we requested them from the secretariat or relevant government officials. The initial list of documents was reviewed with ministry officials to ensure no key documents were left out.

#### Data coding and extraction

All documents underwent a three-stage coding process using MAXQDA. We combined inductive and deductive content analysis, focusing on ‘health equity’ within the document content. First, documents were screened to identify the presence of any of the following elements a) environmental health or climate change and health, b) environmental equity, and/or c) health equity within climate change context. Documents lacking these elements were excluded. Next, the included documents underwent deductive scanning for themes and codes guided by the analysis framework. During this process, health equity considerations were assessed for alignment with principles of participation, distribution, or recognition. Any information not aligning with predefined themes underwent inductive coding.

#### Key words search

Besides using the predeveloped codes in the framework, we actively searched for the explicit mentions of terms and concepts like ‘social justice,’ ‘environmental justice,’ and ‘climate justice,’ which are closely interconnected with health equity. We recorded the frequency and context of these terms to assess the linkages between equity and justice concepts in the policy documents.

#### Synthesis and reporting

All extracted information was synthesized to address the research questions. Additional themes and codes from inductive coding were analyzed to interpret their relation to health equity within the environmental justice framework. A comparative analysis of health and climate change policies was also conducted.

## Results

Out of 33 documents identified, 21 national-level policies, strategies, and plans addressing climate change and/or health in Nepal underwent detailed analysis. These documents were from MoFE [6], MoHP [6], National Planning Commission (NPC) [18], Nepal Law Commission [2], Ministry of Home Affairs [2], and Ministry of Finance [1]. They included three federal documents, four sectoral policies, nine sectoral action plans/strategic frameworks, three sectoral acts, one process documentation, and one standard operating procedure. Six of the 21 documents explicitly addressed health equity within environmental health, while others were included due to their content on climate change and health, or environmental justice. (Supplementary Material S1)

### Conceptualization of health equity in relation to climate change

While there is a positive recognition of climate change impacts on health outcomes, many documents do not explicitly use the term ‘health equity.’ They acknowledge that health disparities stem from social-structural and environmental factors, but the direct link between health equity and climate change is less prominent and, in fact, largely not directly addressed. Health equity is a central focus in most policies of the MoHP either explicitly or implicitly, but not in policies from other entities. Unequal health outcomes related to climate change predominantly manifest as social issues across various cross-cutting sectors from the perspectives of social determinants, environmental determinants, and general vulnerability to climate change and/or disaster events.

The term ‘vulnerable’ is commonly used regarding socio-economic, environmental, and climate vulnerability. SDoH are an overarching approach to explaining the disparities in health outcomes in relation to climate change with documents highlighting various environmental factors (such as seasonal variation, geographic vulnerabilities among others) impacting heath. While there is recognition of the need for tailored policies and interventions, integration strategies are lacking. Nepal’s public health policy emphasizes health equity as a central aim but does not thoroughly address climate change-specific implications. Some documents adopt a rights-based perspective, highlighting the constitutional right to equitable healthcare and a clean environment.

### Use of justice terms

The notions of social, environmental, and climate justice are mentioned across multiple documents aligned with Nepal’s Constitution. ‘Social justice’ appears more frequently, with 27 mentions, notably 21 times in the 15th Plan, five times in the Constitution, and once in Nepal Health Sector Strategic Plan. In contrast both, ‘environmental justice’ and ‘climate justice’ are referenced only twice, indicating lesser emphasis on these terms.

Social justice is a core principle in national documents, encompassing the identification and inclusion of vulnerable groups. Although environmental factors and climate change are included within social justice discussions, they are not distinctly stated. The 15th Plan explicitly links societal well-being with the environment, emphasizing social justice and equality for environmental security. Nepal’s SDG roadmap promotes equal access to economic resources, technology, and basic services to foster an equitable society. The Public Health Policy aims for universal access to high-quality healthcare, including climate change and environmental services, rooted in social justice.

### Interaction between health equity and environmental justice in relation to climate change

Though the term ‘environmental justice’ is infrequent, its principles are recurrent themes across various documents. 16 (Supplementary material S2) of the 21 documents included statements on health equity in relation to the three principles of environmental justice (Supplementary material S3), with or without other environmental justice concerns. Integration stems from the constitutional right to a clean and healthy environment, frequently referenced in most of these documents. The Constitution also briefly mentions intergenerational equity and fair distribution of benefits. One document explicitly included the polluter pays principle without mentioning other environmental justice principles.

#### Distribution

The distribution principle emphasizes the importance of ensuring fairness in distribution of both the environmental benefits and protective measures against environmental harm in policies for achieving environmental justice [22]. However, in the context of Nepal, where resources are limited, the policies tend to focus more on the equitable distribution of natural or financial resources rather than addressing the environmental harms caused by climate change. For instance, climate change policy mandates that 80% of the climate budget be allocated to the local level, however, do not speak about the need to prioritize the vulnerable communities/areas and measures to minimize the environmental health impacts. While many policy statements focus on equitable distribution of natural resource use; the specifics of distribution procedures are less detailed. The health-specific policy documents, in particular, do not specify resource allocation focus for environmental concerns.

Some documents highlight the environmental impacts on vulnerable communities and advocate for legal compensation from polluters. The Environmental Protection Act prohibits activities causing significant harm to public health and the environment. While this Act identifies healthcare facilities as polluters, the SDG roadmap attributes climate change in Nepal to cooking fires, brick kilns, and diesel engines. Despite emphasizing other principles like the polluter pays, detailed costs, and resource allocations for addressing environmental concerns are not specified. The Constitution also mandates environmental impact assessments and equitable distribution of benefits from natural resources or development across federal, provincial, and local levels.

#### Recognition

The recognition principle in environmental justice highlights the disproportionate effects of climate change on different population groups. Documents contain both implicit and explicit references to these disproportionate effects. They recognize Nepal’s vulnerability to climate change and identify specific vulnerable groups such as those in high mountains, marginalized communities, women, indigenous populations, and residents in climate-vulnerable areas. While much of the climate/environmental vulnerability is built on the general overall socio-economic factors, few documents recognize specific vulnerable groups affected by different environmental factors. For analysis, we categorized vulnerabilities mentioned in the documents as general or environmental health specific (Table 1).

**Table 1.** Vulnerabilities described in the policy documents in Nepal.

Factors for vulnerability		Specific vulnerability factors	Vulnerable groups	Health Implication
A) General Vulnerability	Geographic Location	● Floods and inundation, landslides ● Melting of snow surface and glaciers/avalanches ● Earthquakes, fires ● Outbreak of pandemics, epidemics.	● Chure and Terai, Bhitri Madhes and Hilly areas, people and communities who are living along the riverbanks, steep slopes land. Hilly region is at risk of landslides and soil erosion. ● Poles, high mountains of Himalaya region are at risk of avalanche and glacial lake outburst. ● All Nepal being in very active seismic zone, the mountain and Himalayan settlements are at high risk of earthquake	Death and injuries, agricultural production negatively and situation of food insecurity specifically. Scarcity of drinking as well as irrigation water, displacement and destruction of homes, farmland, and other important infrastructures.
	Health sector factors	● Location of health facilities Lack of harmonization between production of HR and their utilization, increasing food security, Lack of basic drinking water and sanitation services, infrastructure built considering country geographic fragility. ● Various types of natural disasters (earthquakes, floods, landslides, fires etc. resulting in the destruction of settlements, infrastructure, and human lives. Climate change and climate variability.	Those affected by climate change- specific communities to be mapped out. Within the Nepali context gender, caste, wealth, ethnicity, and geographic location have been influential factor affecting people’s access to health services.	Rise in water and food related diseases, air pollution, nutrition deficiency related disease, injuries and mental illness, Malaria, Lymphatic filariasis, Japanese encephalitis.
	Socio-economic factors	Disasters like floods, landslides, and fire, heat and cold waves, drought among others	Marginalized and indigenous groups particularly majhi, raute, chepang, satar,those working outside including the poor, women, children and the elderly, children below 5 years of age, girls, and women	Food insecurity, water borne diseases like typhoid, cholera, and other diarrheal diseases
	Availability of resources	Lack of proper irrigation facilities, management of rapid urbanization, fragile infrastructures	VDC, Municipality, village and communities with inadequate and weak quality systems and resources should be categorized as the most vulnerable hotspots, lack of proper settlement arrangements for squatters, tenants, and Guthi farmers; landless Dalit	
B) Environmental Health specific Vulnerability	Seasonal factors	Geophysical and climatic hazards during monsoon	Nationwide. region is at risk of landslides and soil erosion whereasChure and the Terai are at risk of floods, droughts, fire, and epidemics. Himalayan region is at risk of avalanche and glacial lake outburst.	Road accidents, epidemics, earthquake, landslide, flood, snowstorm, droughts related health problems
	Vulnerability analysis	Vulnerability analysis of sensitive hazard like ● GLOFs ● Landslides ● Floods ● Drought	● Some parts of Eastern Himalayan region, as well as Central and the Western higher Mountain clusters ● Hilly and mountain regions ● Almost the entire Terai region ● All the hill and mountain region of the Mid and the Far-western regions	
	Adaptive capacity	Socio-economic condition	The Mid and Far-Western regions and the scattered areas throughout the country are found to be vulnerable.	
	Hydrology and meteorology related indicators	Temperature, precipitation, along with climate induced disasters, landslides, floods, glacier lake outburst, desertification and drying-up of water sources	The mid and far western districts, followed by Western Mountain region and the Central Terai region	Diarrhea, respiratory disease and malaria

General vulnerability factors like geographical location, socio-economic status, gender, caste, or ethnicity, aren’t disputed across various policy areas and are widely discussed. Specific vulnerabilities related to environmental factors outlined in documents are mainly categorized based on factors like geographical location and socio-economic status. Regions such as high mountains, poorer population, marginalized communities, women, landless, indigenous population, vulnerable and disabled individuals, and those in climate vulnerable areas are vulnerable to current and projected climate hazards. However, vulnerability to health is often implicit and needs to be inferred in most documents. General factors for vulnerability in health service access in general are also considered as vulnerability factors for climate change. Only the H-NAP document mentions specific health vulnerabilities from the health sector’s perspective.

Although vulnerability levels vary greatly based on the individual capacity to cope with and to adapt to hazards or crises, i.e., adaptive capacity, [23] this concept is explicitly mentioned in only two documents. Regardless, need for evidence on specific vulnerabilities and impacts is reiterated in multiple policy documents including the health policy, climate change policy and others, highlighting the importance of vulnerability and adaptation assessments in understanding and addressing climate-related challenges.

#### Participation

Public participation in health-system priority setting is believed to enhance accountability, acceptability of the decisions, implementation processes, and consideration of diverse groups [24]. However, among the three principles of environmental justice, participation in climate change and health-related decision-making processes is mentioned the least in policy documents, with only seven of 16 documents mentioning it. Some statements put forward about participation seem more like an acknowledgment of the different stakeholders involved in climate change issues and are by no means sufficient for addressing health equity in the context of climate change decision-making.

Statements on participation principle in climate change vary within sectors. While some documents mention different participatory structures at subnational levels as facilitators for inclusive policy development, their implementation remains unclear. Health-related policies touch on community participation in overall health services but don’t explore their implications in climate change-related decision-making. They also fail to include vulnerable groups or discuss health exclusively. The NAPA mentions participation of women and ‘vulnerable groups’ engagement in NAP formulation process. However, the vulnerability referenced here refers to general overall vulnerability and not specific to health. Likewise, though the Climate Change and Gender Action Plan emphasizes the importance of women’s and marginalized groups’ participation, these statements are made in the context of the effects of disasters and not necessarily the overall climate change or health impacts.

## Discussion

### Systematic conceptualization of health equity in terms of climate change

#### Challenges of defining climate change related health equity

Understanding health equity in terms of environmental health is central to addressing and attaining it. Existing policy documents in Nepal lack a clear definition of health equity in this context and equity-related terms and concepts are inconsistently used with many focusing on disaster events. Moreover, there is also inconsistency in linking climate change with health equity across documents, leading to a lack of consensus on its definition both in general and across sectors. However, the diversity of health equity concerns across different policy areas or challenges to defining it is not unique to Nepal or to climate change as both scientific and policy communities globally lack consensus on the definition and understanding of health equity within environmental health [25,26]. European policies demonstrate a similar disconnect despite the inherent link between health equity and social determinants [3].

This disparity carries significant implications for efforts to address this issue, as many authors have observed that the absence of a shared understanding of health equity and related terminology hinders coordinated policy efforts [27,28]. This challenge is evident in Nepal, where efforts to address health equity within the climate change framework seem fragmented and not necessarily coordinated within sectors. There is thus an urgent need to reconceptualize health equity from a holistic perspective and broaden climate change adaptation and mitigation beyond just disaster management.

### Environmental justice as an overarching goal of climate change response to advance health equity?

It is quite clear that health equity, isn’t an exclusive goal for climate change-related policies in Nepal, but equity in general is a central concern within the framework of environmental justice. Though most of the policy texts do not refer to environmental justice in its deeper meaning, or directly from health equity perspective per se, they do outline principles that align with environmental justice. This is mainly due the constitutional provision guaranteeing the right to a clean and healthy environment, which is frequently referenced in many policy documents. This emphasis can serve as a foundation for understanding health equity in climate change from an environmental justice perspective.

The diversity of environmental justice principles across different policy areas points to different focus areas. Health-related policies prioritize recognizing vulnerable groups for climate change events over equitably distributing resources to these groups. Policies from environment ministries define vulnerability for climate change events but do not focus on health outcomes specifically. As Schlosberg notes that distribution, recognition, and participation are interrelated and interdependent-one cannot pursue one dimension of justice in isolation [22]. Although the principle of recognition has gained more prominence in Nepal compared to the other principles, it sets a foundation for the other two, guiding resource allocation and fair distribution. However, while most policies acknowledge the disproportionate health outcomes of climate change and identify vulnerable groups, there’s a disconnect with health equity.

Distribution, a key principle of environmental justice, is explored to varying extents in policy documents, with a greater focus on resource allocation than on health equity effects. Olsaretti raises three major questions related to the distribution of environmental goods in climate change: who is entitled to what level of protection, who is responsible for bearing the burden of addressing climate change, and how should rights to emit greenhouse gases be distributed [29]. Furthermore, the author identifies three distinct kinds of burden in examining the fair distribution of climate change burdens; a) the cost of mitigation, b) the burden of adaptation, and c) burden/harms on third parties [29]. The policies and strategies in Nepal do not delve into any such detail of distributive justice, and the link between distribution and effects on health equity seems far-fetched indicating that integration is still at a relatively early stage.

The principle of participation plays a prominent role in promoting health equity within the environmental justice framework. Stakeholder participation can enhance the quality of environmental decisions by incorporating more comprehensive information inputs [30]. Friel et al note how empowerment and participation in decision-making is fundamental to building health equity [31]. In the context of climate change, while there is recognition of various vulnerable groups in general and some specific to certain climate change effects, policy statements regarding the necessity for participation are relatively less. This could be attributed to the traditional top-down approach to policymaking [32]. Although specific forums exist at the subnational level to provide affected communities with opportunities to engage in decision-making, and public feedback is solicited via the website, the integration of public feedback into the decision-making process remains unclear. The information presented in the policy documents also seems to portray as what Thomas called as a modified autonomous managerial decision where the manager seeks information from segments of the public but decides alone in a manner which may or may not reflect group influence [33]. If the right to health and to clean environment are to be fully realized climate change needs to have the public health focus. Policies and plans should integrate health equity within environmental justice, emphasizing the need for mainstreaming health equity issues within climate change policies. In this light, considering the work needed to mainstream ‘health equity’ issue within climate change policies, promoting health equity by subsuming within environmental justice appears more feasible. Health equity in the context of climate change cannot be constructed without regard to environmental justice. Addressing health equity in climate change and environment justice needs to go together with efforts to achieve health equity through action in the social determinants. Leveraging the constitutional mandates for a clean environment and universal healthcare access, promoting health equity through environmental justice can foster synergies vital for effective climate responses across the environment and health policy arenas.

## Limitations

Some challenges were encountered during the coding process as disasters were used synonymously with climate change. Also, the health effects of climate change covered a broad spectrum making it difficult to distinguish the indirect effects on health through other sectors. We therefore coded explicit references to health effects while categorizing the implicit references as ‘others’. Additionally, some documents were unofficial translations by international organizations, and English versions were unavailable in some cases, requiring the use of Nepali versions for analysis. To mitigate discrepancies, we back translated the coded content as needed.

## Conclusion

The analysis of policy documents in Nepal through the lens of environmental justice indicates a growing recognition of the interconnectedness between climate change and health equity, urging comprehensive integration across ministries. It was evident that while health sector explicitly uses the term ‘health equity’ in relation to climate change or environment health, policies from MoFE and other national documents approach this issue more from the perspective of environment justice. This suggests that advocating for both health equity and environmental justice simultaneously may be an effective approach to addressing climate change-related health equity, with the environmental justice framework serving as a valuable base for analyzing policies and actions aimed at this goal. Although some policy documents touch on health equity dimensions related to environmental justice principles, they often lack depth. Despite progress in acknowledging the impact of climate change on health equity, challenges persist in fully incorporating health equity into climate change responses. To address this, policymakers must establish a shared understanding of health equity in climate change across sectors, underscoring the importance of fostering collaboration between the health and environmental sectors to address the interconnected challenges posed by climate change. Additionally, more research is needed to identify environmental hazards and vulnerabilities, considering adaptive capacity, to develop more effective and equitable climate change policies, particularly in the context of health.

## Supplementary Material

Updated_Supplementary Material 1 Screening Details_GHA.docx

## Data Availability

All data generated or analyzed during this study are included in this published article [and its supplementary information files]. Any other datasets used and/or analyzed during the current study are available from the corresponding author on reasonable request.

## Abbreviations

GoN: Government of Nepal
H-NAP: Health National Adaptation Plan
MoFE: Ministry of Forest and Environment
MoHP: Ministry of Health and Population
NAPA: National Adaptation Plan of Action
NPC: National Planning Commission
SDG: Sustainable Development Goals
SDoH: Social Determinants of Health
