# Supplementary material for: Examining health equity in Nepal’s climate change and health policies through the lens of environmental justice: insights from a content analysis
Source: Glob Health Action. 2024 Nov 29;17(1):2432069. doi: 10.1080/16549716.2024.2432069 (PMC11610355; doi:10.1080/16549716.2024.2432069)
Supplement: Updated_Supplementary Material 1 Screening Details_GHA.docx [file ZGHA_A_2432069_SM6055.docx]

**Supplementary Material S1: Documents screened for inclusion of health equity**

| **S. No** | **Documents** | **Language** | **Source** | **Type of Document** | **Topics Covered** | | |  |
| --- | --- | --- | --- | --- | --- | --- | --- | --- |
|  |  |  |  |  | Environment Health/ Climate Change and Health | Environmental equity/environment justice | Health equity in the context of Climate change/Environment | DECISION |
| 1 | The Constitution of Nepal, 2016 | English | Nepal Law Commission | Constitution | **×** | **×** |  | INCLUDE |
| 2 | The Fifteenth Plan, (Fiscal Year 2019/20- 2023/24) | English | National Planning Commission | National Strategy | **×** | **×** |  | INCLUDE |
| 3 | National Health Financing Strategy (2021-2031) | English | Ministry of Health and Population | National Strategy |  |  |  | EXCLUDE |
| 4 | Nepal National Health Accounts 2017-2018 | English | Ministry of Health and Population | National Strategy |  |  |  | EXCLUDE |
| 5 | Nepal Sustanaible Development Goals Status and Roadmap: 2016-2030 | English | National Planning Commission | National Strategy | **×** |  |  | INCLUDE |
| 6 | Nepal Public Health Policy 2019 | English | Ministry of Health and Population | Policy | **×** | **×** |  | INCLUDE |
| 7 | The Public Health Service Act, 2075 (2018) | English | Ministry of Health and Population | Act | **×** | **×** |  | INCLUDE |
| 8 | Public Health Service Regulation, 2020 | English (Unofficial translation) | Ministry of Health and Population | Regulation |  |  |  | EXCLUDE |
| 9 | Nepal Health Sector Strategic Plan (2022-2030) | English | Ministry of Health and Population | Strategy | **×** |  | **×** | INCLUDE |
| 10 | National Strategy for reaching the unreached (2016-2030) | Nepali | Ministry of Health and Population | Strategy |  |  |  | EXCLUDE |
| 11 | Urban Health Policy 2072 (2015) | Nepali | Ministry of Health and Population | Policy | **×** |  |  | INCLUDE |
| 12 | Disaster Risk Reduction and Management Act (2017) and Rules (2019) | English | Ministry of Home Affairs | Act | **×** |  |  | INCLUDE |
| 13 | Disaster Risk Reduction National Strategic Plan of Action, 2018 – 2030 | English | Ministry of Home Affairs | Action Plan | **×** | **×** |  | INCLUDE |
| 14 | Nepal Earthquake 2015 Post Disaster Recovery Framework 2016-2020 | English | National Reconstruction Authority | Action Plan |  |  |  | EXCLUDE |
| 15 | National e-health Strategy 2017 | English | Ministry of Health and Population | Strategy |  |  |  | EXCLUDE |
| 16 | Climate change Health National Adaptation Plan (H-NAP) 2017-2021 | English | Ministry of Health and Population | Action Plan | **×** | **×** | **×** | INCLUDE |
| 17 | Nepal National REDD+ Strategy 2018-2022 | English | Ministry of Forest and Environment | Strategy |  |  |  | EXCLUDE |
| 18 | Nepal National Climate Change policy 2019 | English | Ministry of Forest and Environment | Federal Policy | **×** |  |  | INCLUDE |
| 19 | Nepal National Environment Policy 2019 | Nepali | Ministry of Forest and Environment | Policy |  | **×** |  | INCLUDE |
| 20 | The Environment Protection Act 2019 | English | Nepal Law Commission | Act | **×** | **×** |  | INCLUDE |
| 21 | Nepal Environment Protection Regulation | Nepali | Ministry of Forest and Environment | Regulation |  |  |  | EXCLUDE |
| 22 | Climate Change Budget code 2012 | English | National Planning Commission | Process documentation | **×** |  |  | INCLUDE |
| 23 | Climate change financing framework 2017 | English | Ministry of Finance | Framework | **×** |  |  | INCLUDE |
| 24 | National Adaptation Plan (NAP)- 2021-2050 | English | Ministry of Forest and Environment | Federal Action Plan | **×** | **×** | **×** | INCLUDE |
| 25 | National Framework on Local Adaptation Plans for Action (LAPA) 2011 | English | Ministry of Forest and Environment | Local Action Plan | **×** | **×** | **×** | INCLUDE |
| 26 | National framework on climate change induced Loss and Damage 2021 | English | Ministry of Forest and Environment | Framework | **×** | **×** | **×** | INCLUDE |
| 27 | National healthcare waste management standards and operating procedures-2020 | English | Ministry of Health and Population | National Guideline | **×** | **×** |  | INCLUDE |
| 28 | Second Nationally Determined Contributions- (NDC) 2020 | English | Ministry of Forest and Environment | National Action Plan | **×** |  |  | EXCLUDE |
| 29 | NDC Implementation Plan 2023 | Nepali | Ministry of Forest and Environment | National Action Plan | **×** | **×** |  | INCLUDE |
| 30 | Nepal's Long-Term Strategy for Net-Zero Emissions 2021 | English | Ministry of Forest and Environment | National Strategy |  |  |  | EXCLUDE |
| 31 | Climate change Gender Action Plan for the Government of Nepal, 2012 | English |  | National Plan | **×** | **×** | **×** | INCLUDE |
| 32 | Green Climate Fund Engagement Strategy of Nepal | English |  | National Strategy | **×** |  |  | EXCLUDE |
| 33 | Multi-sectoral action plan for prevention and control of NCDs 2021-2025 | Nepali | Ministry of Health and Population | National Action Plan | **×** |  |  | EXCLUDE |
